# Colistin Resistance and Molecular Characterization of the Genomes of *mcr-1*-Positive *Escherichia coli* Clinical Isolates

**DOI:** 10.3389/fcimb.2022.854534

**Published:** 2022-05-06

**Authors:** Qiaoling Li, Changrui Qian, Xueya Zhang, Tingting Zhu, Weina Shi, Mengdi Gao, Chunlin Feng, Ming Xu, Hailong Lin, Li Lin, Junwan Lu, Xi Lin, Kewei Li, Teng Xu, Qiyu Bao, Changchong Li, Hailin Zhang

**Affiliations:** ^1^ The Second Affiliated Hospital and Yuying Children’s Hospital, Wenzhou Medical University, Wenzhou, China; ^2^ Key Laboratory of Medical Genetics of Zhejiang Province, Key Laboratory of Laboratory Medicine, Ministry of Education of China, School of Laboratory Medicine and Life Sciences, Wenzhou Medical University, Wenzhou, China; ^3^ Institute of Biomedical Informatics, School of Laboratory Medicine and Life Sciences, Wenzhou Medical University, Wenzhou, China; ^4^ Institute of Translational Medicine, Baotou Central Hospital, Baotou, China

**Keywords:** *Escherichia coli*, colistin resistance, *mcr-1*, plasmid, novel *mcr-1* variant

## Abstract

Research on resistance against polymyxins induced by the *mcr-1* gene is gaining interest. In this study, using agar dilution method, polymerase chain reaction, and comparative genomic analysis, we investigated the colistin resistance mechanism of clinical *E. coli* isolates. The minimum inhibitory concentration (MIC) analysis results revealed that of the 515 isolates tested, bacteria with significantly increased MIC levels against colistin were isolated in 2019. Approximately one-fifth (17.14% to 19.65%) of the isolates showed MIC values ≥1 mg/L against colistin in 2015, 2016, and 2017. However, in 2019, up to three-quarters (74.11%, 146/197) of the isolates showed MIC values ≥1 mg/L against colistin indicating an increase in colistin resistance. Six isolates (EC7518, EC4968, EC3769, EC16, EC117, EC195, 1.13%, 6/515) were found to carry the *mcr-1* gene and a novel *mcr-1* variant with Met2Ile mutation was identified in EC3769. All six strains showed higher MIC levels (MIC=4 mg/L) than any *mcr-1-*negative strains (MIC ≤ 2 mg/L). Whole-genome sequencing of the six *mcr-1*-positive isolates revealed that EC195 carried the highest number of resistance genes (n = 28), nearly a half more than those of the following EC117 (n = 19). Thus, EC195 showed a wider resistance spectrum and higher MIC levels against the antimicrobials tested than the other five isolates. Multi-locus sequence typing demonstrated that these *mcr-1*-positive strains belonged to six different sequence types. The six *mcr-1* genes were located in three different incompatibility group plasmids (IncI2, IncHI2 and IncX4). The genetic context of *mcr-1* was related to a sequence derived from Tn*6330* (IS*Apl1*-*mcr-1*-*pap2*-IS*Apl1*). Investigations into the colistin resistance mechanism and characterization of the molecular background of the *mcr* genes may help trace the development and spread of colistin resistance in clinical settings.

## Introduction

Polymyxins, a class of non-ribosomal polypeptides characterized by the presence of a lipophilic fatty acyl side chain, were produced by *Bacillus polymyxa* and discovered in the 1940s (Velkov et al., 2013). Due to their neurovirulence and limited renal clearance, polymyxins have been largely abandoned in the clinical treatment of bacterial infections since the 1970s (Ainsworth et al., 1947; Falagas and Kasiakou, 2005; Landman et al., 2008; Hartzell et al., 2009). However, this situation has been changed by the emergence of multidrug-resistant bacteria caused by antibiotic over-consumption (Laxminarayan et al., 2020). Polymyxins, especially polymyxin E (colistin), have been reintroduced as the last recourse against infections caused by gram-negative multidrug-resistant pathogens (Wang Q. et al., 2017). Nonetheless, there is an increasing threat against treatment with polymyxin drugs following the emergence of the plasmid-mediated mobilized colistin resistance (*mcr*) gene in bacteria. It was first identified and characterized in southern China in 2015, denoted as *mcr-1.* To date, ten slightly different variants of the *mcr-1* gene (*mcr-1* to *mcr-10*) have been identified in different bacteria isolated from animals, foods, farms, humans, and the environment (Liu et al., 2016; Sun et al., 2018; Hussein et al., 2021).

The *mcr-1* gene is the most predominant type in MCR family and composed of 1,626 bp in length with a 49% G+C content. It encodes five transmembrane domains and an extracellular catalytic domain. Amino acid sequence analysis revealed that it showed 41% and 40% identities with the lipopolysaccharide export system protein EptA from *Neisseria meningitidis* and phosphoethanolamine (PEA) transfer EptC from *Campylobacter jejuni*, respectively, both of which belong to the PEA transferase family (Hu et al., 2016; Stojanoski et al., 2016). Electrospray mass spectrometry and liquid chromatography-mass spectrometry analyses showed that *mcr-1* possesses biological activity similar to that of PEA transferase (Hinchliffe et al., 2017). It could modulate the lipid A residues of the lipopolysaccharides (LPS), leading to a lower binding affinity of colistin to its target site (Liu et al., 2016; Hussein et al., 2021).

In spite of that the possible mechanism underlying colistin resistance was preliminarily illuminated, the origin, acquisition, emergence, spread pathway and evolutionary lines are not yet completely understood. Epidemiological investigations have found that *mcr-1* rapidly spreads in different ecological niches not only soil, water, wildlife, but also livestock, meat, vegetables, farmland and even humans (Wang et al., 2020; Yang et al., 2021). No less than 10 different bacterial species, including *E. coli*, *Salmonella enterica*, *Klebsiella pneumoniae*, *Enterobacter aerogenes*, *Kluyvera ascorbata*, *Citrobacter freundii*, and *Citrobacter braakii*, as well as more than 10 types of plasmids, including IncX1, IncHI2, IncFIB, IncFIA, IncFII, IncN, IncR, IncQ1, ColpVC, and IncHI1A, participate in mediating its transmission (Wang et al., 2018; Ragupathi et al., 2019). More worryingly, the “superbugs” evolving from *mcr-1* coexisting with other drug resistance genes such as *bla*_NDM-5_ may cause a global challenge to the health care systems. Therefore, more effort should be devoted to putting the ax in the helve (Li et al., 2020; Ling et al., 2020; Xiaomin et al., 2020).

In this study, we investigated the colistin resistance phenotype and prevalence of the *mcr-1* gene among 515 clinical *E. coli* isolates from two tertiary hospitals in Zhejiang Province, China. With the genome sequencing of the *mcr-1*-positive strains, the genetic context of *mcr-1* and the structure of *mcr-1*-harboring plasmids were further analyzed.

## Materials and Methods

### Bacteria Isolation and Species Identification

A total of 515 non-duplicated clinical *E. coli* isolates were collected from two tertiary hospitals in Zhejiang Province, China. Among these isolates, 105, 173, 40, and 197 strains were isolated in 2015, 2016, 2017, and 2019, respectively. Based on the analytical profile index, all strains were identified as *E. coli* using the VITEK 2.0 system (bioMérieux, Durham NC, USA). The six *mcr-1*-positive *E. coli* strains were subsequently subjected to species identification verifying by 16S rDNA sequencing with polymerase chain reaction (PCR) primers (Forward: 5′-AGAGTTTGATCCTGGCTCAG-3′; Reverse: 5′-GGTTACCTTGTTACGACTT-3′), followed by genome sequencing.

### Antimicrobial Susceptibility Testing

The minimum inhibitory concentrations (MICs) of clinical routine antimicrobial agents, including piperacillin (PRL), piperacillin-tazobactam (TZP), ceftazidime (CAZ), ceftriaxone (CTRX), cefoxitin (CFX), cefepime (FEP), imipenem (IMP), meropenem (MEM), aztreonam (AZT), levofloxacin (LEV), ciprofloxacin (CIP), amikacin (AMK), gentamicin (GEN), chloramphenicol (CAP) and rifampicin (RIF) were determined by the agar dilution method. The MICs of colistin were determined by the broth microdilution in cation-adjusted Mueller–Hinton broth (CAMHB) (Gajdács et al., 2020). We interpreted the results in accordance with the Clinical and Laboratory Standards Institute (CLSI) guidelines (CLSI, 2020). *E. coli* ATCC 25922 was used as the standard reference strain.

### Conjugation Experiment

The transferability of *mcr-1* was tested by conjugation experiment with *mcr-1*-positive *E. coli* as donors and rifampicin-resistant *E. coli* C600 as a recipient. The MacConkey agar plates containing rifampicin (512 µg/mL) and colistin (1 µg/mL) were used to select *mcr-1*-positive transconjugants. PCR analysis of *mcr-1* and antimicrobial susceptibility testing were carried out to confirm the transconjugants.

### Polymerase Chain Reaction (PCR)

All *E. coli* strains were screened for the presence of the mcr-1 gene by PCR using primers (Forward: 5′-CGGTCAGTCCGTTTGTTC-3′, Reverse: 5′-CTTGGTCGGTCTGTA GGG-3′) (Liu et al., 2016). The PCR products were purified and sequenced by Sanger sequencing (Generay, Shanghai, China).

### Next-Generation Sequencing and Bioinformatics Analysis

Genomic DNA of six *mcr-1*-harboring *E. coli* strains was extracted using Bacterial Genomic DNA Miniprep kit (Generay, Shanghai, China), and subsequently sequenced by Illumina HiSeq 2500 (Illumina, Inc., San Diego, CA, United States). The short reads were assembled using SPAdes software (version3.14.0). EC195 showed the widest resistance spectrum and highest MIC levels against all the antimicrobials tested in this study, while the other five strains exhibited similar MIC levels, so we chose EC195 and one of the other five EC7518 as the representative isolates for whole genome sequencing. These two isolates were further sequenced by Pacific Bioscience (PacBio) RSII systems at the Shanghai Personal Biotechnology Co., Ltd. (Shanghai, China). Then, hybrid assembly was performed using Unicycler v0.4.8, with both short and long reads. The assembled sequences were annotated using Prokka (Seemann, 2014) and then corrected by BLAST (Boratyn et al., 2013) searches against the UniProtKB/Swiss-Prot, RefSeq, ISfinder (Siguier et al., 2006), and CARD (Jia et al., 2017) databases. The multi-locus sequence typing (MLST) and plasmid replicon type (Inc groups) identification were conducted using MLST (https://pubmlst.org/) (Jolley and Maiden, 2010) and PlasmidFinder (https://cge.cbs.dtu.dk//services/PlasmidFinder/), respectively. The core genome phylogenetic tree was generated using kSNP3 (Gardner et al., 2015), and plasmid maps were generated using CGView (Grant and Stothard, 2008). Gene organization diagrams were generated using Python script and modified with Inkscape 1.0 (https://inkscape.org/).

### Statistical Analyses

The differences in the colistin MIC distribution of 509 *mcr-1*-negative *E. coli* isolates among the four years were tested using the χ^2^ test. A P value <0.05 was considered to be statistically significant. All analyses were conducted using the SPSS software (version 22.0; SPSS Inc., Chicago, IL, USA).

### Sequence Data Availability

The genome data of *mcr-1*-positive strains have been submitted to NCBI under the BioProject accession number PRJNA770868. The mcr-1.34 gene in this work have been submitted to GenBank under accession numbers MZ450868.

## Results and Discussion

### Colistin MIC of the Strains

**Figure 1:**
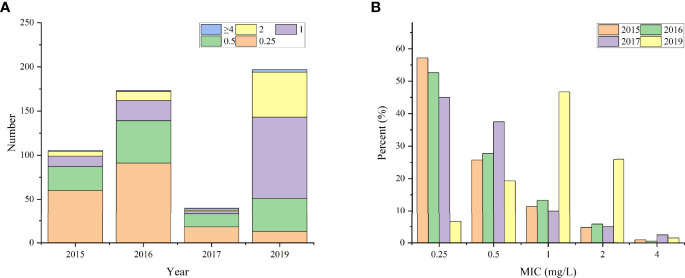
The distribution of clinical isolates with different MIC values. **(A)** the total number; **(B)** the percentage.

Approximately 17.14% (18/105), 19.65% (34/173), 17.5% (7/40), and 74.11% (146/197) of the isolates showed MIC values ≥1 mg/L against colistin in 2015, 2016, 2017 and 2019, respectively. However, only six isolates showed MIC values of 4 mg/L, including three strains isolated in 2019 and one each in 2015, 2016, and 2017 (**Figure 1A**).

Further analysis demonstrated that isolates from 2015, 2016, and 2017 exhibited similar percentage of MIC to colistin, ranging from 0.25 mg/L to 2 mg/L. The percentages of different MIC levels were 53.65% (169/315), 28.57% (90/315), 12.38% (39/315), and 5.40% (17/315) at 0.25, 0.5, 1, and 2 mg/L, respectively. In 2019, nearly half (47.42%, 92/194) of the strains had a MIC value of 1 mg/L, and approximately 26.29% (51/194) of the strain had a MIC of up to 2 mg/L. Moreover, the MIC value of 0.25 mg/L was found in only 6.7% (13/194) of the bacteria isolated in 2019, which was just one-eighth (53.65%) of that from the previous years (**Figure 1B**). This might be associated with the increase in use of polymyxin to treat bacterial infections in humans (http://app1.nmpa.gov.cn/data_nmpa/face3/dir.html), which contributes to the emergence and increase of polymyxin-resistant bacteria.

### *mcr-1* Gene Detection and Sequencing

In this study, six (1.17%) *mcr-1-*positive *E. coli* were identified. Previous publications reported *mcr-1* positive rates of 0.35% (12/3434), 0.69% (2/291), and 0.6% (4/700) in clinical *E. coli* isolates from Chinese hospitals between 2002 and 2016 (Lu et al., 2018), in 2018 (Liao et al., 2020), and from 2014 to 2015 (He et al., 2017), respectively. One study reported an *mcr-1* positive rate of 0.87% (6/689) in clinical *Salmonella* spp. between 2009 and 2018 (Fan et al., 2020). A higher *mcr-1*-positive rate of 3.5% (24/688) in *E. coli* was reported from 2016 to 2018 in Guangzhou, China (Shen et al., 2020). These results revealed the increasing prevalence of the *mcr-1* gene in clinical settings.

Of the six *mcr-1*-positive strains, EC3769, EC4968, and EC7518 were isolated in 2015, 2016, and 2017, respectively, whereas EC16, EC117, and EC195 were isolated in 2019. The results showed that the six *mcr-1*-harboring strains exhibited higher MICs against colistin than the negative strains. All six *mcr-1* positive strains found in this study were resistant to colistin with a MIC value of 4 mg/L, which was in accordance with previous studies (He et al., 2017; Liao et al., 2020). Furthermore, all *mcr-1*-positive strains showed resistance to multiple antimicrobial agents, exhibiting similar MIC levels against 14 antimicrobials tested, except MEM and IMP (**Table 1**). One of the *mcr-1*-positive isolates, namely, EC195, showed the broadest resistance spectrum and highest MIC levels against all 14 antimicrobials tested in this study, especially for MEM and IMP. EC195 showed MIC levels >8 mg/L to MEM and IMP, approximately ≥16-fold higher than the other five strains, which showed MIC ≤ 1 mg/L to either MEM or IMP (**Table 1**).

**Table 1:** MIC values for original isolates and their *mcr-1*-positive transconjugants (mg/L).

Isolates Antibiotic Agents	ATCC25922	EC7518	EC4968	EC3769	EC16	EC117	EC195	EC7518/EC600	EC4968/EC600	EC16/EC600	EC117/EC600	EC3769/EC600	EC600
**COL** >	**0.25** >	**4** >	**4** >	**4** >	**4** >	**4** >	**4** >	**2** >	**2** >	**2** >	**2** >	**1** >	**0.25** >
**PRL** >	**4** >	**256** >	**>256** >	**>256** >	**>256** >	**>256** >	**>256** >	**<2** >	**<2** >	**<2** >	**<2** >	**<2** >	**<2** >
**TZP** >	**4** >	**32** >	**32** >	**32** >	**64** >	**32** >	**>128** >	**8** >	**8** >	**8** >	**8** >	**16** >	**2** >
**CAZ** >	**0.5** >	**16** >	**32** >	**16** >	**16** >	**16** >	**>32** >	**0.5** >	**0.5** >	**0.5** >	**0.5** >	**0.5** >	**0.25** >
**CFX** >	**4** >	**16** >	**16** >	**32** >	**8** >	**16** >	**>256** >	**16** >	**16** >	**16** >	**16** >	**16** >	**8** >
**CTRX** >	**0.125** >	**>32** >	**>32** >	**32** >	**>32** >	**32** >	**>32** >	**<0.06** >	**<0.06** >	**<0.06** >	**<0.06** >	**0.125** >	**<0.06** >
**FEP** >	**0.125** >	**16** >	**>32** >	**16** >	**16** >	**16** >	**>32** >	**0.06** >	**0.06** >	**0.06** >	**0.06** >	**0.125** >	**0.06** >
**IMP** >	**0.25** >	**1** >	**0.5** >	**0.5** >	**0.5** >	**0.5** >	**32** >	**0.125** >	**0.125** >	**0.125** >	**0.125** >	**0.125** >	**<0.06** >
**MEM** >	**0.06** >	**0.125** >	**0.125** >	**0.125** >	**0.125** >	**0.06** >	**16** >	**<0.03** >	**<0.03** >	**<0.03** >	**<0.03** >	**<0.03** >	**<0.03** >
**AZT** >	**0.25** >	**64** >	**64** >	**16** >	**64** >	**16** >	**128** >	**0.25** >	**0.25** >	**0.25** >	**0.25** >	**0.25** >	**<0.125** >
**LEV** >	**0.06** >	**>16** >	**>16** >	**>16** >	**>16** >	**>16** >	**>16** >	**0.25** >	**0.25** >	**0.25** >	**0.25** >	**0.5** >	**0.25** >
**CIP** >	**0.03** >	**>16** >	**>16** >	**>16** >	**16** >	**>16** >	**>16** >	**0.125** >	**0.125** >	**0.125** >	**0.125** >	**0.25** >	**0.125** >
**AMK** >	**4** >	**64** >	**16** >	**16** >	**64** >	**16** >	**16** >	**1** >	**1** >	**1** >	**1** >	**1** >	**1** >
**GEN** >	**1** >	**>32** >	**32** >	**>32** >	**>32** >	**32** >	**>32** >	**<0.125** >	**<0.125** >	**<0.125** >	**<0.125** >	**<0.125** >	**<0.125** >
**CAP** >	**8** >	**512** >	**256** >	**256** >	**256** >	**256** >	**512** >	**4** >	**4** >	**4** >	**4** >	**4** >	**4** >
**RIF** >	**<1** >	**4** >	**4** >	**8** >	**4** >	**4** >	**8** >	**>1024** >	**>1024** >	**>1024** >	**>1024** >	**>1024** >	**>1024** >

COL, colistin; PRL, piperacillin; TZP, piperacillin tazobactam; CAZ, ceftazidime; CTRX, cefatriaxone; CFX, cefoxitin; FEP, cefepime; IMP, imipenem; MEM, meropenem; AZT, aztreonam; LEV, levofloxacin; CIP, ciprofloxacin; AMK, amikacin; GEN, gentamicin; CAP chloramphenicol; RIF, rifampicin.

Sequencing the *mcr-1* gene revealed that five of the six isolates exhibited 100% identity to the primary reported *mcr-1* gene (KP347127). By contrast, the *mcr-1* gene of EC3769 was a novel variant (designated mcr-1.34 in this study) with a one-point mutation at nucleotide position 6 (G to A), which led to the substitution of methionine residue to isoleucine residue (Met to Ile) in comparison with the MCR-1.1 protein (AKF16168). The MCR-1 protein contains an N-terminal inner membrane-bound domain (residues 1–241) and a C-terminal soluble catalytic domain (residues 215–541) (Gao et al., 2016). The substitution of the novel variant in this study was very close to the start of the transmembrane domain.

### Whole-Genome Sequencing of *mcr-1*-Positive Strains

**Figure 2:**
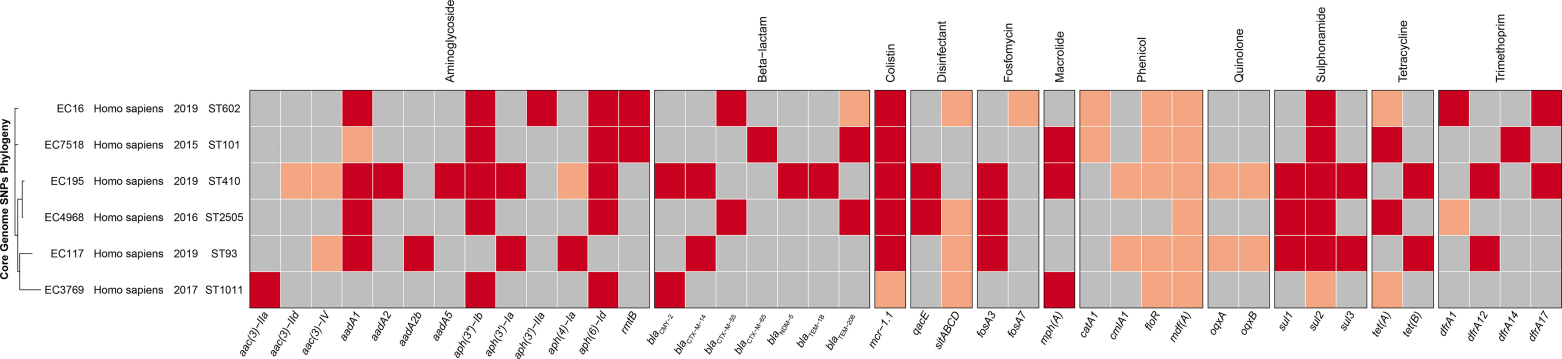
The phylogenetic tree and annotation of epidemiological and genomic features of six *mcr-1*-positive strains. The heatmap is used to display the types of acquired antimicrobial resistance genes. The presence in identity (red) or variant (pink) and absence (gray) of antimicrobial resistance genes are indicated.

The general features of the genomes of the six *mcr*-positive isolates (four draft genomes of EC3769, EC117, EC16, and EC4968 and two complete genomes of EC195 and EC7518) are summarized in **Table 2**. *In silico* analysis revealed that these strains belonged to different MLST, including ST1011 (EC3769), ST93 (EC117), ST101 (EC7518), ST602 (EC16), ST410 (EC195), and ST2505 (EC4968) (**Figure 2**). ST1011 was proposed to be closely related to the poultry sector, identified in Lebanon and duck farms in southeast coastal China (Wang et al., 2021). However, an *mcr-1*-carrying *E. coli* ST1011 has also been isolated from a fecal sample of a 21-year-old male patient (Liang et al., 2021). Even though ST101 has been reported as one of the most prevalent sequence types (STs) among *bla*_NDM_-positive *E. coli* strains in poultry production, it has been found in hospital sewage water and is regarded as an infection-causing isolate (Wang Y. et al., 2017; Jin et al., 2018). In addition, ST410 has been frequently reported in animal husbandry-related epidemiology studies (Wang et al., 2020; Cheng et al., 2021).

**Table 2:** General features of the genomes of the *mcr-1*-positive *E. coli* strains.

	Size (bp)	G+C (%)	ORFs	MLST/Inc Type
**EC195** (complete genome)				
Chromosome	4,915,634	50.51	4582	ST410
pEC195-MCR-1	253,380	46.80	282	IncHI2/IncN
pEC195-93	93,120	52.03	101	IncFIA/IncFIB/IncFII/IncQ1
pEC195-2	2,088	47.27	2	–
**EC7518** (complete genome)				
Chromosome	4,941,244	50.55	4596	ST101
pEC7518-111	111,696	46.41	135	IncFIB
pEC7518-MCR-1	63,359	42.97	81	IncI2
pEC7518-49	49,706	52.59	58	IncFII
pEC7518-2	2621	46.47	2	–
**EC16** (incomplete genome)	5,228,642	–	–	ST602 IncFIB/IncFII/IncI2/IncN/IncX1
**EC117** (incomplete genome)	5,264,248	–	–	ST93 IncFIA/IncFIB/IncFIC/IncHI2/IncI2
**EC4968** (incomplete genome)	5,245,949	–	–	ST2505 IncFIB/IncFIC/IncFII/IncI1-I/IncI2/IncQ1
**EC3769** (incomplete genome)	5,147,491	–	–	ST1011 IncFIB/IncFIC/IncFII/IncX4

ST93 was previously regarded as a similar ST to ST410; however, an isolate from the blood sample of an 84-year-old man in a Uruguay hospital with resistance to third-generation cephalosporins changed that perception (Papa-Ezdra et al., 2020). ST93 may pose a more significant threat to human health than ST410. ST602 is identified in animal lesion organs (Liu et al., 2021), whereas *E. coli* ST2505 is a novel ST carrying *mcr-1* gene first reported in this study. However, some prevalent STs of *mcr-1* carrying *E. coli* in previous publications such as ST10, ST131, and ST156 (He et al., 2017) were not found in this study, especially *E. coli* ST10, the most prevalent ST of hospital-associated *E. coli* (Shen et al., 2018).

Up to 41 different antibiotic resistance genes (ARGs) were identified in these *mcr-1*-carrying *E. coli* genomes, including β-lactam resistance genes, aminoglycoside, tetracycline, and sulfonamide genes (**Figure 2**). Moreover, *aad*A1, *aad*A2, *aad*A5, and the efflux genes *oqx*A and *oqx*B were also identified. This enabled them to develop multidrug resistance phenotypes. According to previous reports, *bla*_CTX-M-65_, *bla*_TEM-206_, *bla*_CMY-2_, *bla*_NDM-5_, *bla*_CTX-M-14_, *bla*_TEM-1B_, and *bla*_CTX-M-55_ belong to extended-spectrum β-lactamases, the main problem of *E. coli* drug resistance, which can decrease the activity of β-lactam ring and cause bacterial resistance to antimicrobials (Cullik et al., 2010; Smet et al., 2012). Genes s*ul1*, *sul2*, and *sul3* contribute to alleviating the damage of *E. coli* caused by sulfonamide, especially *sul2*, which is usually located in multiple resistance-determining regions of a large transferable plasmid and can thus spread along with its carrier plasmid (Enne et al., 2001). The acetyltransferase genes, including *aac(3)-IIa*, *aac(3)-Iv*, *aac(3)-IId*, and the phosphotransferase genes, including *aph(6)-Id*, *aph(3′)-Ib*, *aph(4)-Ia*, *aph(3′)-Ia*, and *aph(3′)-IIa*, are thought to increase *E. coli* resistance by changing the target position and decreasing medicine efficacy (Recht and Puglisi, 2001). Furthermore, since its first report in pig manure, in 2003, many studies have demonstrated that *oqx*A and *oqx*B are related to diversified drug resistance by extracellularly extruding antimicrobial poison (Sørensen et al., 2003; Hansen et al., 2007; Sato et al., 2011).

Among the six *mcr-1*-carrying *E. coli* genomes, EC195 carried the highest number (28) of resistance genes, nearly a half more than those (19) of the following EC117. Resistance genes such as *aadA2*, *aac(3)-IIa*, *aadA5*, and *bla*_NDM-5_ only appeared in EC195, which might contribute to its broader resistance spectrum and higher MIC levels against the antimicrobials tested than those of the other five isolates. The resistance genotype of EC195 was consistent with the phenotype. It had 12 classes of resistance genes and showed resistance to the corresponding antimicrobials (**Table 1**). Moreover, the specific resistance against MEM and IMP of EC195 might be related to the presence of *bla*_NDM-5_, the carbapenem resistance-related gene only identified in EC195.

### Characterization and Comparative Genomic Analysis of the *mcr-1*-Harboring Plasmids

In the six *mcr-1*-positive genomes, the *mcr-1* genes were all located on three types of incompatibility (Inc) group plasmids, IncHI2 (pEC195-MCR-1), IncI2 (pEC16-MCR-1, pEC117-MCR-1, pEC4968-MCR-1, and pEC7518-MCR-1), and IncX4 (pEC3769-MCR-1). Five (exclusive of pEC195-MCR-1) of the six *mcr-1* carrying plasmids were transferable and the transconjugants were obtained. The transconjugants showed colistin MIC levels slightly lower than those of their corresponding original isolates (**Table 1**). In addition, complete plasmid sequences carrying *mcr-1* were retrieved from GenBank for further analysis. IncHI2 was the most common Inc type among them, followed by IncI2 and IncX4 (**Supplementary Figure S1**). This result was consistent with a previous report, which showed that IncI2, IncHI2, and IncX4 accounted for more than 90% of the plasmids reported to carry *mcr-1* (Berglund et al., 2018).

**Figure 3:**
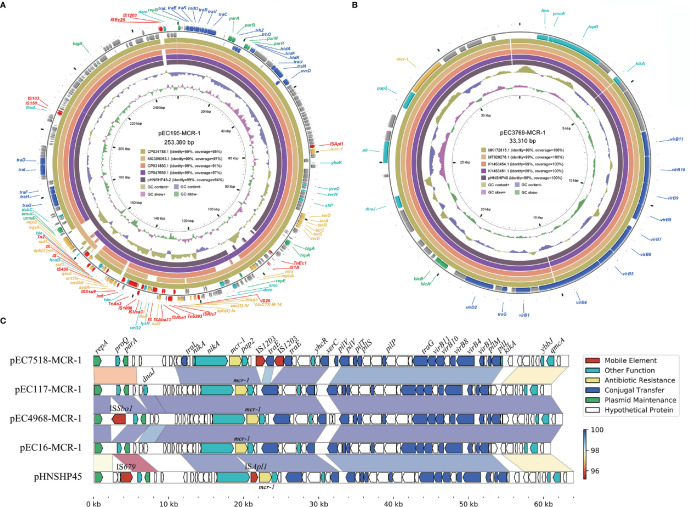
Diagram of the six *mcr-1*-harboring plasmids. **(A)** the plasmid map of pEC195-MCR-1; **(B)** the plasmid map of pEC3769-MCR-1; **(C)** Comparison of the four IncI2 plasmids and pHNSHP45. Genes are denoted by arrows and are colored based on gene function classification. Regions of > 95% identity are marked by gradient colors.

The IncHI2 plasmid pEC195-MCR-1 was 253,380 bp in length with a G+C content of 46.80%. It shared 99% nucleotide identity and over 95% coverage with plasmid pHNSHP45-2 (KU341381), which was the first reported IncHI2 plasmid carrying *mcr-1* (Arcilla et al., 2016) (**Figure 3A**). The variable region of pEC195-MCR-1 consisted of a group of resistance genes including *bla*_CTX-M-14_, *fosA3*, *aac(3)-Iv*, *aph(4’)-Ia*, *sul2*, *floR*, *aadA*, *cmlA6*, *ant3Ia*, *sul3*, *aph(4’)-Ia*, *sul1*, *oqxAB*, a tellurium resistance gene cluster and *mcr-1*. However, additional ARGs (*mrx*/*mphA*) and mobile genetic elements (MGEs; IS*1203*, IS*Ec25*, IS*1A*, and Tn*Ec1*) were identified in pEC195-MCR-1. The IncHI2-type plasmid has been reported to be the most diverse plasmid that contains a large MDR region composed of various ARGs and MGEs (Li et al., 2017). Co-occurrence of *mcr-1* and other ARGs, especially EBSL, in plasmids can cause difficulties in clinical antibacterial treatment.

IncX4 plasmid pEC3769-MCR-1 was 33,310 bp in length and had an average G+C content of 41.58% (**Figure 3B**). The plasmid was almost identical (100% coverage, 99% identity) to other *mcr-1*-harboring IncX4 plasmids such as pHNSHP49 (MF774188) (Wu et al., 2018). These IncX4 plasmids have no known ARGs other than *mcr-1*. However, self-transmissible IncX4-type plasmids are important vehicles responsible for disseminating the *mcr-1* gene among Enterobacteriaceae worldwide (Fernandes et al., 2016; Wang Q. et al., 2017).

The length of the four IncI2 plasmids ranged from 60,960 to 63,359 bp (**Figure 3C**). These plasmids consisted of replication genes, horizontal transfer, maintenance, and stability region and only carried one ARG *mcr-1*. BLASTN results revealed they were similar to the first reported *mcr-1*-harboring IncI2 plasmid pHNSHP45 (NZ_KP347127). These IncI2 plasmids share a highly conserved backbone and contain minor differences in the *repA* neighboring region. The IncI2-type plasmid was the earliest reported vector of the plasmid-mediated *mcr-1* gene, which plays a significant role in rapidly mobilizing and acquiring *mcr-1* (Liu et al., 2016; Petrillo et al., 2016; Zelendova et al., 2021).

### Genetic Contexts of *mcr-1*

**Figure 4:**
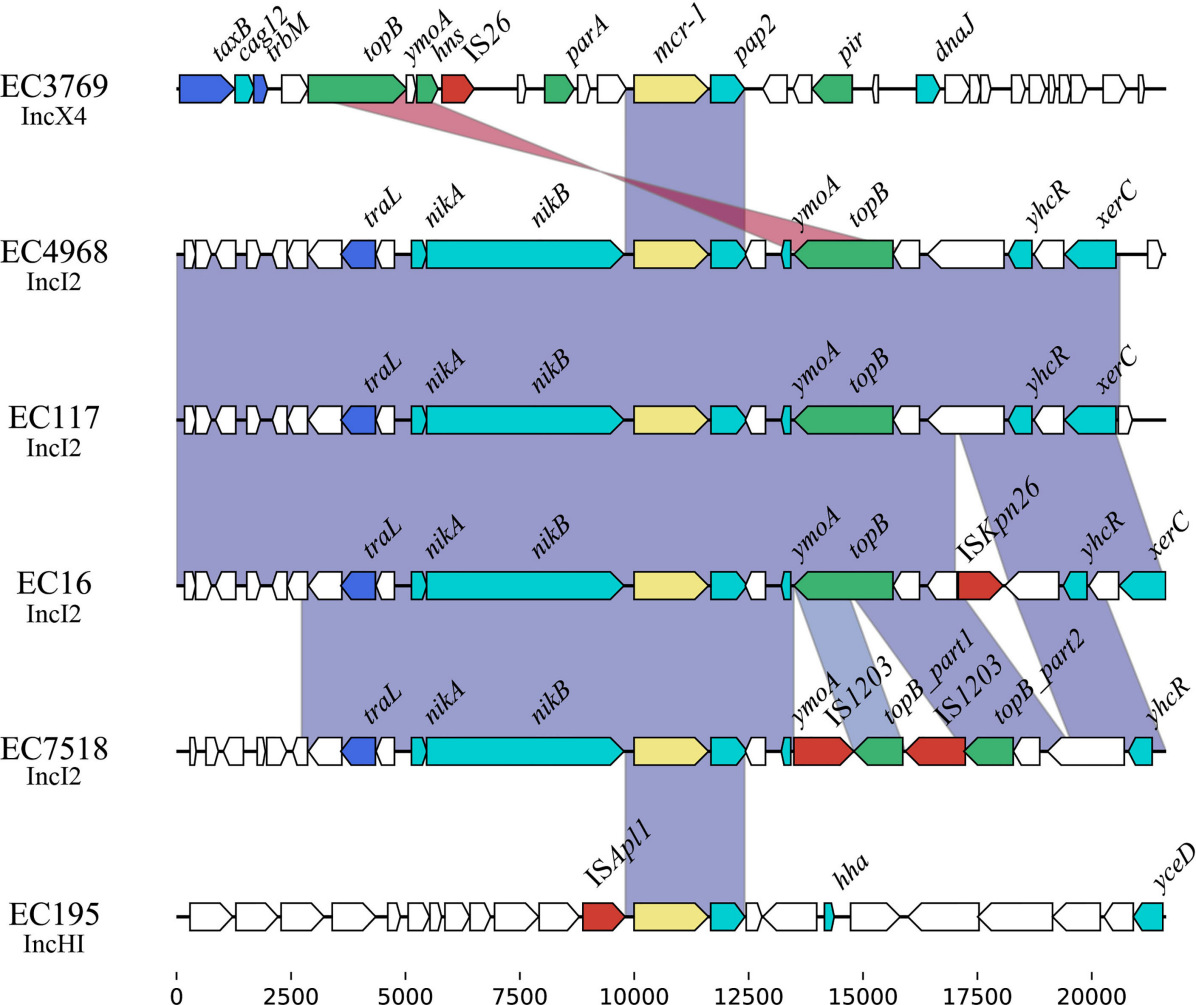
Genetic context of the *mcr-1* genes on the sequenced genomes.

Comparative genomic analysis was performed on the ~20-kb sequences around the *mcr-1* genes. The results revealed that an ~2.6-kb region encoding *mcr-1*-*pap2* was conserved among these plasmids (**Figure 4**), which was in accordance with previous reports about the *mcr-1* gene (Poirel et al., 2016). This 2.6-kb region is always associated with the IS*Apl1* element and can be mobilized in the form of Tn*6330* (IS*Apl1*-*mcr-1*-*pap2*-IS*Apl1*) (Snesrud et al., 2018). However, among these sequenced *mcr-1*-bearing plasmids, only pEC195-253K carried a single copy of IS*Apl1* beside *mcr-1*, which indicated that *mcr-1* was stable on these plasmids. Among these four IncI2 plasmids, the *mcr-1* gene was located between *tral*-*nikA*-*nikB* and *ymoA*-*topB* sequences. In addition, these regions of pEC4968 and pEC117 were almost identical, but two copies of IS*1203* truncated the ymoA-topB genes of pEC7518-63K. IS*26* flanked by an 8-bp direct repeat was identified upstream of *mcr-1* on pEC3769-MCR-1, but was not related to the mobilization of the *mcr-1* context (Manageiro et al., 2019).

Furthermore, the 5-kb region around the *mcr-1* gene of IncHI2, IncI2, and IncX4 plasmids was retrieved from GenBank of NCBI and subsequently clustered with a threshold identity >95% (**Supplementary Figure S1**). A complete Tn*6330* transposon was identified on IncHI2 and IncI2 plasmids, demonstrating its role in the initial acquisition of *mcr-1*. It has been reported that the *nikA*-*nikB*-*mcr-1*-*pap2* structure is conserved among IncI2 plasmids (Feng et al., 2019). Herein, we also found that *nik*B upstream of *mcr-1* in all IncI2 plasmids was often truncated to different lengths. Furthermore, in Cluster 6 of IncI2 plasmids, we noticed that the *pap2* gene, which encodes the PAP2-family protein to facilitate the transfer of *mcr-1*, was lost.

## Conclusion

Although a series of *mcr* genes [*mcr*_(1-10)_] were detected and reported in succession, *mcr-1*, the first member of the MCR family discovered in 2015, is still recognized as a critical factor in polymyxin resistance. In this study, we screened *mcr-1* in 515 human clinical *E. coli* isolates and found 1.17% (6/515) *mcr-1-*positive strains, and these strains showed higher resistance to colistin (with MIC levels of 4 mg/L) than the *mcr-1*-negative strains (all with MIC levels <4 mg/L). We identified 41 ARGs in the 6 *mcr-1* positive strains, of which EC195 not only carried the highest number (28) of resistance genes but also exhibited a broader resistance spectrum and higher MIC levels. Furthermore, the unique resistance phenotype against MEM and IMP might be related to the rare identification of *bla*_NDM-5_ from the EC195 genome. MLST found that these six *mcr-1-*positive strains belonged to six different STs. A novel *mcr-1* variant was identified in EC3769 with a one-point mutation at nucleotide position 6, causing an amino acid variation. These findings may provide a new perspective on the molecular characteristics of resistance caused by *mcr-1*.

## Data Availability Statement

The datasets presented in this study can be found in online repositories. The names of the repository/repositories and accession number(s) can be found in the article/**Supplementary Material**.

## Author Contributions

HZ, CL, and QB designed the study. HL, CF, TZ, QL, and XZ acquired data. CQ, CF, WS, LL, MG, MX, and JL performed the results analysis and interpreted data. QL, CQ, and QB wrote the first draft of the paper. KL, HZ, XL, and TX revised it critically for important intellectual content. All co-authors approved the final version.

## Funding

This study was supported by the National Natural Science Foundation of China (81973382, 81960381 and 81700011), Zhejiang Provincial Natural Science Foundation of China (LQ17H010003, LY19C060002 and LQ17H190001), and the Science and Technology Project of Wenzhou City, China (N20210001).

## Conflict of Interest

The authors declare that the research was conducted in the absence of any commercial or financial relationships that could be construed as a potential conflict of interest.

## Publisher’s Note

All claims expressed in this article are solely those of the authors and do not necessarily represent those of their affiliated organizations, or those of the publisher, the editors and the reviewers. Any product that may be evaluated in this article, or claim that may be made by its manufacturer, is not guaranteed or endorsed by the publisher.
