# Supplementary material for: Colistin Resistance and Molecular Characterization of the Genomes of mcr-1-Positive Escherichia coli Clinical Isolates
Source: Front Cell Infect Microbiol. 2022 May 6;12:854534. doi: 10.3389/fcimb.2022.854534 (PMC9120429; doi:10.3389/fcimb.2022.854534)
Supplement: Supplementary file 1 [file DataSheet_1.docx]

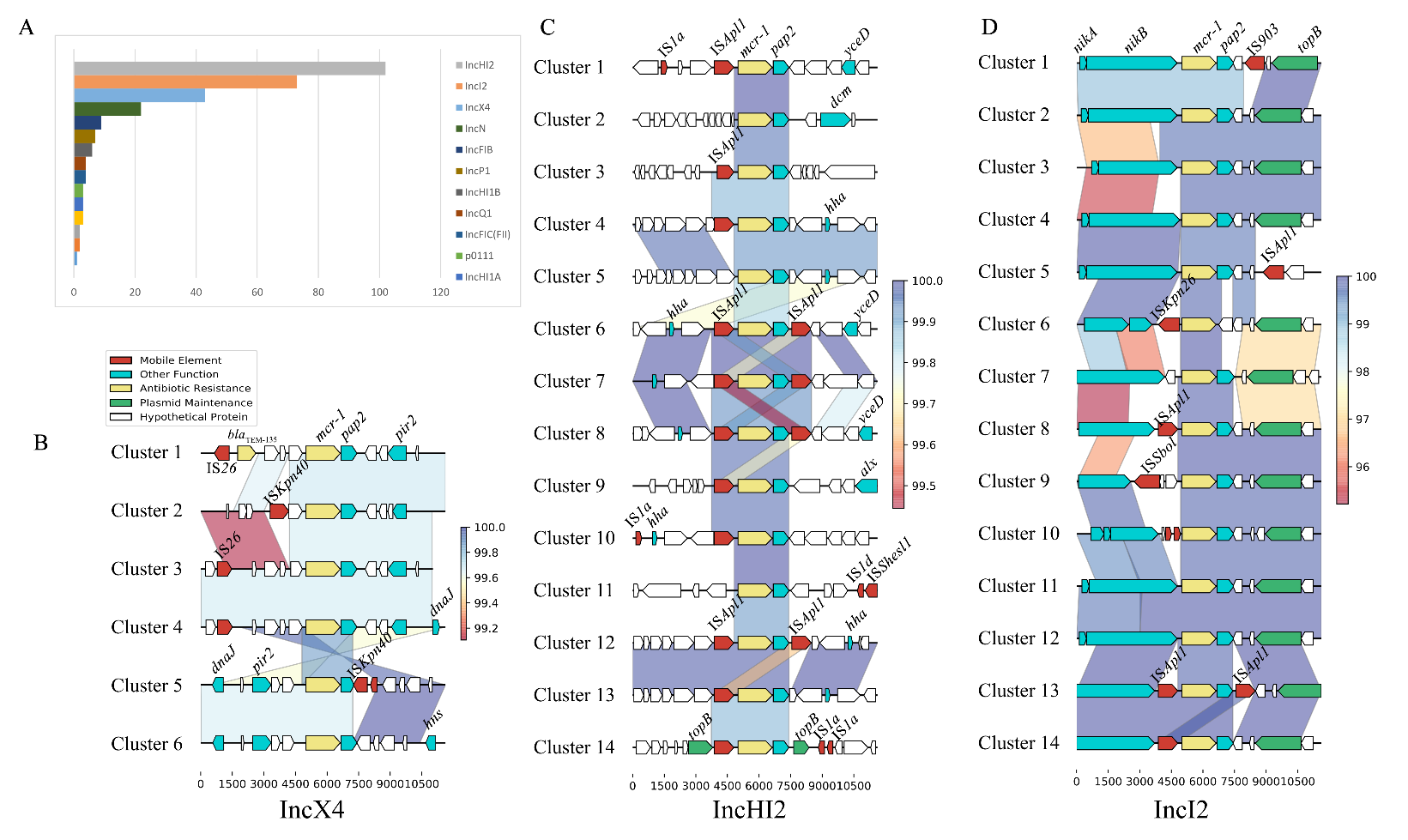


**Figure S1. Statistics of Inc group of *E. colimcr-1*-positive plasmid (A) and structure of the *mcr-1* gene-related regions (BCD).**The5-kb region around the mcr-1 gene of IncX4**(B)**, IncHI2**(C)** and IncI2**(D)**plasmids were clustered at 95% identity using the cd-hit program. Representative sequences from each clusterare shown. The arrows represent sequence units or genes and are color coded, with the arrowheadsindicating the direction of transcription. Gene names are indicated above arrows, and sequence units of unknown function or orthologous regions are left blank.
