# Supplementary material for: Colistin Resistance and Molecular Characterization of the Genomes of mcr-1-Positive Escherichia coli Clinical Isolates
Source: Front Cell Infect Microbiol. 2022 May 6;12:854534. doi: 10.3389/fcimb.2022.854534 (PMC9120429; doi:10.3389/fcimb.2022.854534)
Supplement: Supplementary file 2 [file Table_1.docx]

**Table S1. Accession number of *mcr-1*-positive plasmids of *E. coli* used in this study**

| **Accession number** | **Inc group** | **mcr-1 context Cluster** |
| --- | --- | --- |
| NZ_JACYGC010000002.1 | IncHI2, IncHI2A, IncQ1 | IncHI2 Cluster 1 |
| NZ_JACYGD010000003.1 | IncHI2, IncHI2A | IncHI2 Cluster 10 |
| CP020493.1 | IncHI2, IncHI2A, IncQ1 | IncHI2 Cluster 10 |
| CP019356.1 | IncHI2, IncHI2A, IncN | IncHI2 Cluster 11 |
| CP019360.1 | IncFIB, IncFIC(FII), IncHI2, IncHI2A | IncHI2 Cluster 12 |
| CP022169.1 | IncHI2, IncHI2A | IncHI2 Cluster 12 |
| CP032993.1 | IncHI2, IncHI2A | IncHI2 Cluster 12 |
| CP019353.1 | IncHI2, IncHI2A, IncN | IncHI2 Cluster 12 |
| CP031850.1 | IncHI2, IncHI2A | IncHI2 Cluster 13 |
| CP038454.1 | IncHI2, IncHI2A | IncHI2 Cluster 13 |
| CP019214.3 | IncHI2, IncHI2A, IncN | IncHI2 Cluster 13 |
| CP026492.1 | IncHI2, IncHI2A, IncN | IncHI2 Cluster 13 |
| CP034788.1 | IncHI2, IncHI2A, IncN | IncHI2 Cluster 13 |
| CP038139.1 | IncHI2, IncHI2A, IncN | IncHI2 Cluster 13 |
| CP042587.1 | IncHI2, IncHI2A, IncN | IncHI2 Cluster 13 |
| CP047659.1 | IncHI2, IncHI2A, IncN | IncHI2 Cluster 13 |
| CP047666.1 | IncHI2, IncHI2A, IncN | IncHI2 Cluster 13 |
| CP047877.1 | IncHI2, IncHI2A, IncN | IncHI2 Cluster 13 |
| CP071135.1 | IncHI2, IncHI2A, IncN | IncHI2 Cluster 13 |
| CP040806.1 | IncHI2, IncHI2A, IncQ1 | IncHI2 Cluster 13 |
| CM008267.1 | IncFIB, IncHI2, IncHI2A | IncHI2 Cluster 14 |
| CP050131.1 | IncHI2, IncHI2A | IncHI2 Cluster 2 |
| CP061116.1 | IncHI2, IncHI2A | IncHI2 Cluster 2 |
| CP034390.1 | IncHI2, IncHI2A | IncHI2 Cluster 3 |
| CP033224.2 | IncHI2, IncHI2A | IncHI2 Cluster 4 |
| CP047116.1 | IncHI2, IncHI2A | IncHI2 Cluster 4 |
| CP033347.2 | IncHI2, IncHI2A | IncHI2 Cluster 5 |
| CP072803.1 | IncHI2, IncHI2A | IncHI2 Cluster 5 |
| CP027202.2 | IncHI2, IncHI2A, IncN | IncHI2 Cluster 5 |
| CP059836.1 | IncHI2, IncHI2A, IncN | IncHI2 Cluster 5 |
| CP026936.2 | IncHI2, IncHI2A | IncHI2 Cluster 6 |
| CP026933.2 | IncHI2, IncHI2A, IncQ1 | IncHI2 Cluster 7 |
| CP023143.1 | IncHI2, IncHI2A | IncHI2 Cluster 8 |
| CP019394.1 | IncHI2, IncHI2A | IncHI2 Cluster 9 |
| CP021209.1 | IncHI2, IncHI2A | IncHI2 Cluster 9 |
| CP029215.1 | IncHI2, IncHI2A | IncHI2 Cluster 9 |
| CP033351.2 | IncHI2, IncHI2A | IncHI2 Cluster 9 |
| CP034820.1 | IncHI2, IncHI2A | IncHI2 Cluster 9 |
| CP035916.1 | IncHI2, IncHI2A | IncHI2 Cluster 9 |
| CP035918.1 | IncHI2, IncHI2A | IncHI2 Cluster 9 |
| CP061123.1 | IncHI2, IncHI2A | IncHI2 Cluster 9 |
| CP061125.1 | IncHI2, IncHI2A | IncHI2 Cluster 9 |
| CM008264.1 | IncFIB, IncFIC(FII), IncFII, IncHI2A, IncN | IncHI2 Undefined |
| NZ_WYDM02000005.1 | IncHI2 | IncHI2 Undefined |
| CM008265.1 | IncHI2, IncHI2A | IncHI2 Undefined |
| CM008266.1 | IncHI2, IncHI2A | IncHI2 Undefined |
| CP022165.1 | IncHI2, IncHI2A | IncHI2 Undefined |
| CP022735.1 | IncHI2, IncHI2A | IncHI2 Undefined |
| CP025402.1 | IncHI2, IncHI2A | IncHI2 Undefined |
| CP034785.1 | IncHI2, IncHI2A | IncHI2 Undefined |
| CP058311.1 | IncHI2, IncHI2A, IncN | IncHI2 Undefined |
| CP073361.1 | IncHI2, IncHI2A, IncN | IncHI2 Undefined |
| CP069705.1 | IncI2(Delta) | IncI2 Cluster 1 |
| CM012292.1 | IncI2(Delta) | IncI2 Cluster 10 |
| AP019686.1 | IncI2 | IncI2 Cluster 11 |
| AP018806.1 | IncI2 | IncI2 Cluster 12 |
| AP018812.1 | IncI2 | IncI2 Cluster 12 |
| CM015672.1 | IncI2 | IncI2 Cluster 12 |
| CP016405.1 | IncI2 | IncI2 Cluster 12 |
| CP019052.1 | IncI2 | IncI2 Cluster 12 |
| CP021194.1 | IncI2 | IncI2 Cluster 12 |
| CP029059.1 | IncI2 | IncI2 Cluster 12 |
| CP030769.1 | IncI2 | IncI2 Cluster 12 |
| CP032081.1 | IncI2 | IncI2 Cluster 12 |
| CP041921.1 | IncI2 | IncI2 Cluster 12 |
| CP044025.1 | IncI2 | IncI2 Cluster 12 |
| CP047014.1 | IncI2 | IncI2 Cluster 12 |
| CP049356.1 | IncI2 | IncI2 Cluster 12 |
| CP051226.1 | IncI2 | IncI2 Cluster 12 |
| CP061186.1 | IncI2 | IncI2 Cluster 12 |
| CP066368.1 | IncI2 | IncI2 Cluster 12 |
| CP072908.1 | IncI2 | IncI2 Cluster 12 |
| NZ_JADGML010000002.1 | IncI2 | IncI2 Cluster 12 |
| CP021176.1 | IncI2(Delta) | IncI2 Cluster 12 |
| CP022452.1 | IncI2(Delta) | IncI2 Cluster 12 |
| CP024139.1 | IncI2(Delta) | IncI2 Cluster 12 |
| CP029184.1 | IncI2(Delta) | IncI2 Cluster 12 |
| CP033254.2 | IncI2(Delta) | IncI2 Cluster 12 |
| CP033355.2 | IncI2(Delta) | IncI2 Cluster 12 |
| CP043036.1 | IncI2(Delta) | IncI2 Cluster 12 |
| CP047579.1 | IncI2(Delta) | IncI2 Cluster 12 |
| CP050290.1 | IncI2(Delta) | IncI2 Cluster 12 |
| CP055257.1 | IncI2(Delta) | IncI2 Cluster 12 |
| CP055260.1 | IncI2(Delta) | IncI2 Cluster 12 |
| CP058303.1 | IncI2(Delta) | IncI2 Cluster 12 |
| CP070924.1 | IncI2(Delta) | IncI2 Cluster 12 |
| NZ_WLVM01000008.1 | IncI2(Delta) | IncI2 Cluster 12 |
| NZ_WLVN01000011.1 | IncI2(Delta) | IncI2 Cluster 12 |
| AP018110.1 | IncI2 | IncI2 Cluster 13 |
| AP017614.1 | IncI2 | IncI2 Cluster 14 |
| AP017619.1 | IncI2 | IncI2 Cluster 14 |
| AP017622.1 | IncI2 | IncI2 Cluster 14 |
| CP019264.1 | IncI2 | IncI2 Cluster 14 |
| CP024135.1 | IncI2 | IncI2 Cluster 14 |
| CP024142.1 | IncI2 | IncI2 Cluster 14 |
| CP024148.1 | IncI2 | IncI2 Cluster 14 |
| CP026644.1 | IncI2 | IncI2 Cluster 14 |
| CP030284.1 | IncI2 | IncI2 Cluster 14 |
| CP030766.1 | IncI2 | IncI2 Cluster 14 |
| CP031296.1 | IncI2 | IncI2 Cluster 14 |
| CP031550.1 | IncI2 | IncI2 Cluster 14 |
| CP032940.1 | IncI2 | IncI2 Cluster 14 |
| CP034400.1 | IncI2 | IncI2 Cluster 14 |
| CP034405.1 | IncI2 | IncI2 Cluster 14 |
| CP041113.1 | IncI2 | IncI2 Cluster 14 |
| CP041926.1 | IncI2 | IncI2 Cluster 14 |
| CP042471.2 | IncI2 | IncI2 Cluster 14 |
| CP019254.1 | IncI2(Delta) | IncI2 Cluster 14 |
| CP020548.1 | IncI2(Delta) | IncI2 Cluster 14 |
| CP025679.1 | IncI2(Delta) | IncI2 Cluster 14 |
| CP032990.1 | IncI2(Delta) | IncI2 Cluster 14 |
| CP034109.1 | IncI2(Delta) | IncI2 Cluster 14 |
| CP061128.1 | IncI2(Delta) | IncI2 Cluster 2 |
| CP060519.1 | IncI2 | IncI2 Cluster 3 |
| CP055253.1 | IncI2(Delta) | IncI2 Cluster 4 |
| CP045521.1 | IncI2(Delta) | IncI2 Cluster 5 |
| CP024156.1 | IncI2 | IncI2 Cluster 6 |
| CP019277.1 | IncI2(Delta) | IncI2 Cluster 7 |
| CP018106.1 | IncI2 | IncI2 Cluster 8 |
| CP018112.1 | IncI2 | IncI2 Cluster 8 |
| CP018118.1 | IncI2 | IncI2 Cluster 8 |
| CP018124.1 | IncI2 | IncI2 Cluster 8 |
| CP015913.1 | IncI2(Delta) | IncI2 Cluster 9 |
| CP021205.1 | IncI2 | IncI2 Undefined |
| CP032069.1 | IncI2 | IncI2 Undefined |
| NZ_JACYGH010000005.1 | IncX4 | IncX4 Cluster 1 |
| CP042607.1 | IncX4 | IncX4 Cluster 2 |
| CP033357.1 | IncX4 | IncX4 Cluster 3 |
| CM007714.1 | IncX4 | IncX4 Cluster 4 |
| CM008162.1 | IncX4 | IncX4 Cluster 4 |
| CP015977.1 | IncX4 | IncX4 Cluster 4 |
| CP017246.1 | IncX4 | IncX4 Cluster 4 |
| CP018773.2 | IncX4 | IncX4 Cluster 4 |
| CP024132.1 | IncX4 | IncX4 Cluster 4 |
| CP024149.1 | IncX4 | IncX4 Cluster 4 |
| CP027257.1 | IncX4 | IncX4 Cluster 4 |
| CP028174.1 | IncX4 | IncX4 Cluster 4 |
| CP030795.1 | IncX4 | IncX4 Cluster 4 |
| CP032076.1 | IncX4 | IncX4 Cluster 4 |
| CP033349.2 | IncX4 | IncX4 Cluster 4 |
| CP034833.1 | IncX4 | IncX4 Cluster 4 |
| CP037906.1 | IncX4 | IncX4 Cluster 4 |
| CP038181.1 | IncX4 | IncX4 Cluster 4 |
| CP042644.1 | IncX4 | IncX4 Cluster 4 |
| CP045460.1 | IncX4 | IncX4 Cluster 4 |
| CP046418.1 | IncX4 | IncX4 Cluster 4 |
| CP046719.1 | IncX4 | IncX4 Cluster 4 |
| CP047664.1 | IncX4 | IncX4 Cluster 4 |
| CP048826.1 | IncX4 | IncX4 Cluster 4 |
| CP053735.1 | IncX4 | IncX4 Cluster 4 |
| CP059291.1 | IncX4 | IncX4 Cluster 4 |
| CP060524.1 | IncX4 | IncX4 Cluster 4 |
| CP063489.1 | IncX4 | IncX4 Cluster 4 |
| CP069716.1 | IncX4 | IncX4 Cluster 4 |
| CP072316.1 | IncX4 | IncX4 Cluster 4 |
| LT838201.1 | IncX4 | IncX4 Cluster 4 |
| NZ_JACYGB010000004.1 | IncX4 | IncX4 Cluster 4 |
| NZ_JACYGF010000017.1 | IncX4 | IncX4 Cluster 4 |
| CM016895.1 | IncX4 | IncX4 Cluster 5 |
| CM016890.1 | IncX4 | IncX4 Cluster 6 |
| CM016893.1 | IncX4 | IncX4 Cluster 6 |
| CP019072.1 | IncX4 | IncX4 Cluster 6 |
| CP024041.1 | IncX4 | IncX4 Cluster 6 |
| CP024462.1 | IncX4 | IncX4 Cluster 6 |
| CP024919.1 | IncX4 | IncX4 Cluster 6 |
| CP049122.1 | IncX4 | IncX4 Cluster 6 |
| CP063335.1 | IncX4 | IncX4 Cluster 6 |
| CP031291.1 | IncX4 | IncX4 Undefined |
| CP041641.1 | IncC |  |
| CP063474.1 | IncFIA(HI1), IncHI1A, IncHI1B |  |
| CP070915.1 | IncFIA(HI1), IncHI1A, IncHI1B |  |
| CP033094.1 | IncFIA(HI1), IncHI1A, IncHI1B, IncN |  |
| CP029748.1 | IncFIB, IncFIC(FII), IncN |  |
| CP041997.1 | IncFIB, IncFIC(FII), IncN |  |
| CP044377.1 | IncFIB, IncHI1B, IncN |  |
| CP044386.1 | IncFIB, IncHI1B, IncN |  |
| CP065953.1 | IncFIB, IncHI1B, IncN |  |
| CP029493.1 | IncFIB, p0111 |  |
| CP047381.1 | IncFII |  |
| NZ_JABFON010000003.1 | IncI1-I(Alpha) |  |
| NZ_JABFOO010000006.1 | IncI1-I(Alpha) |  |
| AP021897.1 | IncP1 |  |
| CP021078.1 | IncP1 |  |
| CP024128.1 | IncP1 |  |
| CP032987.1 | IncP1 |  |
| CP063470.1 | IncP1 |  |
| CP063478.1 | IncP1 |  |
| CP063495.1 | IncP1 |  |
| CP035313.1 | p0111 |  |
| CP047090.1 | p0111 |  |
